# Global trends in research of neuroimmune in lung diseases over past decade: a bibliometric analysis

**DOI:** 10.3389/fimmu.2026.1751834

**Published:** 2026-03-05

**Authors:** Yongyan Zhao, Chunfei Li, Yuhong Cheng, Wenjing Dai

**Affiliations:** 1 Department of Respiratory and Critical Care Medicine, The First Affiliated Hospital, Chengdu Medical College, Chengdu, China; 2 Key Laboratory of Geriatric Respiratory Diseases of Sichuan Higher Education Institute, Chengdu, China; 3 Key Specialty: 2025 Sichuan Province Provincial Clinical Key Specialty Construction Project, Chengdu, China

**Keywords:** neuroimmunity, lung diseases, bibliometrics, CiteSpace, VOSviewer, cross-database validation

## Abstract

**Background:**

The neuroimmune axis plays a crucial role in maintaining pulmonary homeostasis and influencing disease outcomes. Although significant progress has been made in this interdisciplinary field, the research remains scattered, and a unified understanding is still lacking. This study applies bibliometric techniques to delineate the knowledge landscape, examining its dynamics and structural features. The objective is to establish a systematic framework for fostering disciplinary consensus and directing future research directions.

**Methods:**

Data for the primary bibliometric analysis and visualization were drawn from the Science Citation Index Expanded within the Web of Science Core Collection. Visualization was performed using VOSviewer and CiteSpace. To ensure the robustness and validity of our findings, a complementary dataset was retrieved from PubMed for cross-database comparative analysis. The WoS dataset served as the basis for the main analytical and visual mapping processes, while the PubMed dataset was utilized to verify the consistency of key trends and patterns.

**Results:**

Using the WoS Core Collection, 2,171 publications were analyzed. Annual output rose steadily over the past decade, peaking at 280 articles in 2022 (R² = 0.9315). The United States led with 699 publications, an H−index of 81 and 39.12 average citations, followed by China (n=547). At the institutional level, the University of California system was most productive (n=62), while Harvard University showed the greatest impact (4,652 total citations, H−index=31); collaboration analysis revealed a core-periphery network centered on Harvard Medical School, with Shanghai Jiao Tong and Fudan University increasing their collaborative activity. Research hotspots centered on neuroimmunity, notably neuropeptides and autonomic regulation in asthma, and have expanded post−COVID to neuro−pulmonary complications. Cross−database validation with PubMed (1,970 articles) confirmed high consistency in publication trends and core topics.

**Conclusion:**

This study is the first to systematically analyze the knowledge structure and developmental trajectory of neuroimmunology in pulmonary diseases from 2015 to 2024. The analysis clarifies the leading position of USA and the rise of China and other emerging contributors. Cross−database validation supports the robustness of these findings. Collectively, these results deepen comprehension of the field’s knowledge framework and furnish empirical guidance for future research priorities and allocation of resources.

## 1 Introduction

The lungs, as an important physiological barrier for direct interaction between the human body and the external environment, are continuously exposed to various foreign substances, pathogens, and irritants (1). Traditionally, the immune system and the nervous system have been regarded as two independently functioning defense systems: the immune system provides specific protection through cellular and molecular mechanisms, while the nervous system is responsible for rapidly detecting threats and coordinating the body’s responses (2). However, groundbreaking research in recent years has revealed a profound and complex bidirectional dialogue between these two systems, highlighting the cellular and molecular mechanisms underlying this interaction. Soluble mediators including neurotransmitters, neuropeptides, and cytokines broadly mediate these interactions by signaling through cell surface receptors on target cells (3). The nervous system, through its densely distributed peripheral nerve endings in the airways and lung parenchyma, can almost instantaneously detect environmental changes, such as chemical, mechanical, and thermal stimuli. Sensory and autonomic neurons, together with released neuropeptides (such as substance P, CGRP) and classical neurotransmitters (such as acetylcholine, norepinephrine), have been shown to engage in direct, specific crosstalk with innate immune effectors (for example alveolar macrophages, mast cells, neutrophils and dendritic cells) as well as adaptive immune populations (including CD4+ and CD8+ T lymphocytes and B cells), thereby modulating their migration, activation status, and downstream effector functions (4). This neuro-immune interplay is crucial for preserving pulmonary homeostasis, coordinating host defense responses, and promoting tissue repair (5). When this finely tuned regulatory network is disrupted, the protective responses can turn into pathological processes. Abnormally persistent neuroimmune signaling is recognized as a principal driver of multiple chronic pulmonary disorders (6).Understanding the specific molecular mechanisms of neuro-immune interactions not only provides a new perspective for elucidating the pathophysiology of lung diseases but also opens up unprecedented therapeutic prospects. The pandemic has altered the global burden trends of chronic respiratory diseases (CRD), particularly asthma, followed by chronic obstructive pulmonary disease (COPD) (7). With the global aging population, the burden trends associated with low social demographic index (SDI) levels have raised concerns (8). As COVID-19 becomes endemic, the elderly will be affected by repeated viral infections, which will increasingly manifest in the coming years (7). Consequently, investigating how neuro-immune crosstalk influences therapeutic outcomes in pulmonary diseases is essential.

Bibliometric analysis is a multidisciplinary approach that has been utilized in gynecology, orthopedics, gastroenterology, complementary and alternative medicine, and other clinical specialties (9, 10). Through assessment of databases and literature features, bibliometric methods can predict trajectories of scientific publications and act as a practical tool to identify research frontiers. Moreover, they yield robust metrics to guide experimental designs and funding allocation (11). To date, no bibliometric analysis has specifically examined the role of neuro-immune interactions in pulmonary diseases. This review will provide a systematic account of neuro-immune regulation in lung disorders and perform a bibliometric assessment to comprehensively map the development trends and major research hotspots in this field. It will also address practical obstacles to clinical translation and outline concrete future directions.

## 2 Methodology

### 2.1 Data sources and search strategies

**Figure 1:**
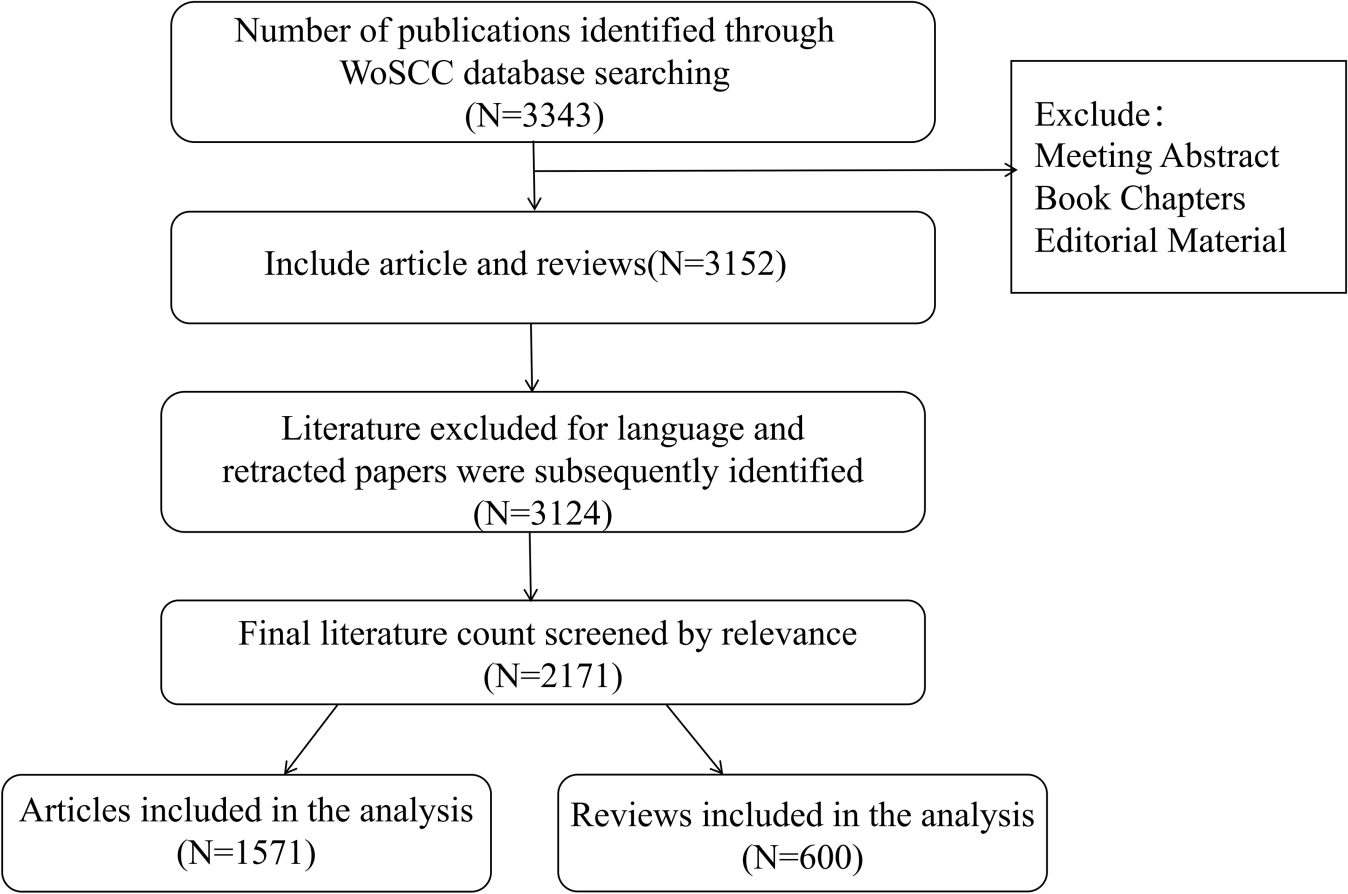
Screening procedure for eligible studies.

Data for this study were retrieved from two independent databases—the Web of Science (WOS) Core Collection and PubMed—to conduct a robust bibliometric analysis and cross-database validation. The publication period was restricted from 2015 to 2024, in both databases. All searches and data downloads were completed on a single day to avoid discrepancies caused by daily database updates. The primary dataset for analytical mapping and visualization was sourced from the Science Citation Index Expanded (SCI-Expanded) within the Web of Science Core Collection (WOSCC). The search strategy for WOS was as follows: TS=(((“neuro-immune” OR neuroimmune OR “neuro-immun*” OR neuroimmun* OR “neuro-inflamm*” OR neuroinflamm*) AND (lung OR pulmonary OR airway OR respirat*))OR(((“autonomic nervous system” OR “sympathetic nervous system” OR “parasympathetic nervous system” OR “vagus nerve” OR “cholinergic”) AND (immun* OR inflamm*)) AND (lung OR pulmonary OR respirat*))OR((“neurogenic inflammation” OR “neurogenic pulmonary” OR “neuropeptide” OR “substance P” OR “CGRP”) AND (lung OR pulmonary OR airway))OR((“lung-brain axis” OR “pulmonary-brain axis” OR “neuro-immune axis” OR “neuroimmune axis”) AND (lung OR pulmonary))). As of December 31, 2024, all publications were retrieved, resulting in 3,343 relevant documents. After excluding 19 non-English publications and 9 retracted articles, and limiting the document type to articles and reviews to ensure research quality, a total of 3124 documents were included. Final 2,171 documents screened by relevance (**Figure 1**). We employed CiteSpace to detect and remove duplicate publications, then extracted titles, abstracts, keywords, and country affiliations, resulting in a dataset of 1,571 articles and 600 reviews for analysis. To ensure the robustness of our findings and mitigate potential database-specific biases, a parallel validation dataset was retrieved from PubMed using the following conceptually equivalent search strategy, which combines Medical Subject Headings (MeSH) and title/abstract keywords: ((“Neuroimmunomodulation”[MeSH] OR “neuro-immune” OR neuroimmune OR “neuro-immun*” OR “neuroinflammation”[tiab] OR “neuro-inflammatory”[tiab]) AND (“Lung”[MeSH] OR “Respiratory System”[MeSH] OR lung OR pulmonary OR airway* OR respirat*)) OR (((“Autonomic Nervous System”[MeSH] OR “Sympathetic Nervous System”[MeSH] OR “Parasympathetic Nervous System”[MeSH] OR “Vagus Nerve”[MeSH] OR “vagus nerve” OR cholinergic) AND (“Immunity”[MeSH] OR immun* OR inflamm*)) AND (“Lung”[MeSH] OR pulmonary OR respirat*)) OR ((“Neurogenic Inflammation”[MeSH] OR “neurogenic inflammation”[tiab] OR “neurogenic pulmonary”[tiab] OR “Neuropeptides”[MeSH] OR “neuropeptide*”[tiab] OR “Substance P”[MeSH] OR “substance P”[tiab] OR “Calcitonin Gene-Related Peptide”[MeSH] OR “CGRP”[tiab]) AND (“Lung”[MeSH] OR pulmonary OR airway*)) OR (((“Brain”[MeSH] AND “Lung”[MeSH]) OR “lung-brain axis”[tiab] OR “pulmonary-brain axis”[tiab] OR “neuro-immune axis”[tiab] OR “neuroimmune axis”[tiab]) AND (“Lung”[MeSH] OR pulmonary)). Retrieval was restricted to English-language records within the same period and further limited to study types including Adaptive Clinical Trial, Clinical Study, Clinical Trial, Meta-Analysis, Network Meta-Analysis, Review, and Systematic Review. All searches were conducted on the same day, and results were downloaded in plain text format to ensure consistency and reproducibility. Ultimately, 1,970 publications were included for the comparative validation analysis.

### 2.2 Mapping tools

For this analysis, VOSviewer (v.1.6.20), and CiteSpace (v.6.4.R1) were employed to process all 2,171 documents. VOSviewer is a program for constructing and visualizing maps from network data (12). Through citation data analysis, the tool constructs co–citation, collaboration, and citation networks, offering a lucid visual depiction of the knowledge structure within a given research domain. VOSviewer (v.1.6.20) was employed to map and visualize countries, institutions, journals, and authors (13). CiteSpace is a Java-based tool for analyzing and visualizing network data (Chen, 2006). It remains a distinctive and widely cited program in information visualization research (14). It is widely used in academic research and trend forecasting. Therefore, in this study, we utilized VOSviewer to map coupling relationships among authors, journals, and countries, and applied CiteSpace to visualize the knowledge base and hotspots of neuroimmune research in lung diseases while forecasting emerging research frontiers.

The number of publications and citation counts are often used as indicators of bibliometrics. As two important perspectives for measuring research significance, the number of publications (Np) is commonly used to quantify productivity, while citation counts (Nc) can reflect impact (15).

The H−index is mainly employed to assess a researcher’s scholarly output and to forecast future research impact. It merges productivity and influence by establishing a threshold that links Np and Nc. A researcher with H publications, each cited at least H times, is assigned an H−index equal to H. Moreover, although the H−index was developed to evaluate an individual’s scholarly performance, it can be adapted to quantify the publication output of a country or region, and likewise the productivity of an institution or a journal (16).

Furthermore, the impact factor (IF), as computed from the latest edition of the Journal Citation Reports (JCR), is widely regarded as one of the principal indicators of the scholarly quality and influence of medical journals (17). The Global Citation Score (GCS) is regarded as an article’s Nc metric. It serves as a key measure of a paper’s contribution to its discipline, where a high GCS reflects substantial attention from researchers around the world (18).

VOSviewer (v.1.6.20) serves to construct and visualize bibliometric network maps, offering analytical tools to display relationships among entities like authors, journals, and keywords, thereby enhancing understanding of the academic landscape (11, 13). In this study, VOSviewer is applied for co-citation and co-occurrence analyses: node size corresponds to publication volume, line thickness denotes relationship intensity, and node color distinguishes clusters or time periods.

CiteSpace applies multiple approaches including cluster analysis, timeline/distribution profiling, dual−map overlays, and citation−burst detection to generate visual maps of knowledge domains and their developmental trajectories. These approaches help uncover principal themes, nascent topics, and how research in a given field has evolved (19). Cluster analysis can classify keywords and uncover fundamental research themes related to neuroimmunology in pulmonary diseases. By frequently utilizing a large number of keywords and references, it helps identify emerging research trends. This approach facilitates a comprehensive understanding of how different aspects of neuroimmunology are related to lung disease research.

### 2.3 Key parameter settings of bibliometric tools

CiteSpace and VOSviewer were employed to perform bibliometric analyses and visualization. For CiteSpace, the time span was set to 2015–2024 with a time slice of 1 year. Node selection used the g−index (k = 5); additional settings were Link Retaining Factor (LRF) = 2.5, maximum links per node (L/N) = 10, Look−back years (LBY) = 5, and topology pruning coefficient (e) = 1.0. Cluster analysis was conducted using the log−likelihood ratio (LLR) algorithm. Network size was reported by N (number of nodes) and E (number of edges). Cluster quality was evaluated by modularity Q and the weighted mean silhouette S, with Q > 0.3 and S > 0.7 regarded as indicative of a significant and reliable clustering structure.

VOSviewer was applied sequentially to visualize networks for countries, institutions, journals, and co−cited references. For each analysis type, appropriate minimum occurrence thresholds and Attraction/Repulsion parameter values were chosen based on the underlying data distribution. Normalization was performed using fractional counting, similarity between items was calculated with the cosine measure, and clustering utilized the software’s default density−based clustering algorithm (13). In the network visualizations, node size was proportional to item frequency and label size correlated with total link strength; spatial layout was generated using the VOS core layout algorithm to ensure clear and interpretable maps.

## 3 Results

### 3.1 Overview of research on neuroimmunology in the field of lung diseases

This study analyzed 2,171 papers from 88 countries and 2,960 institutions, authored by 12,675 researchers, published across 855 journals. These papers cited references from 11,150 journals, encompassing a total of 129,933 citations. When evaluating the development status and research interest of a discipline, the number of publications remains the most critical indicator.

**Figure 2:**
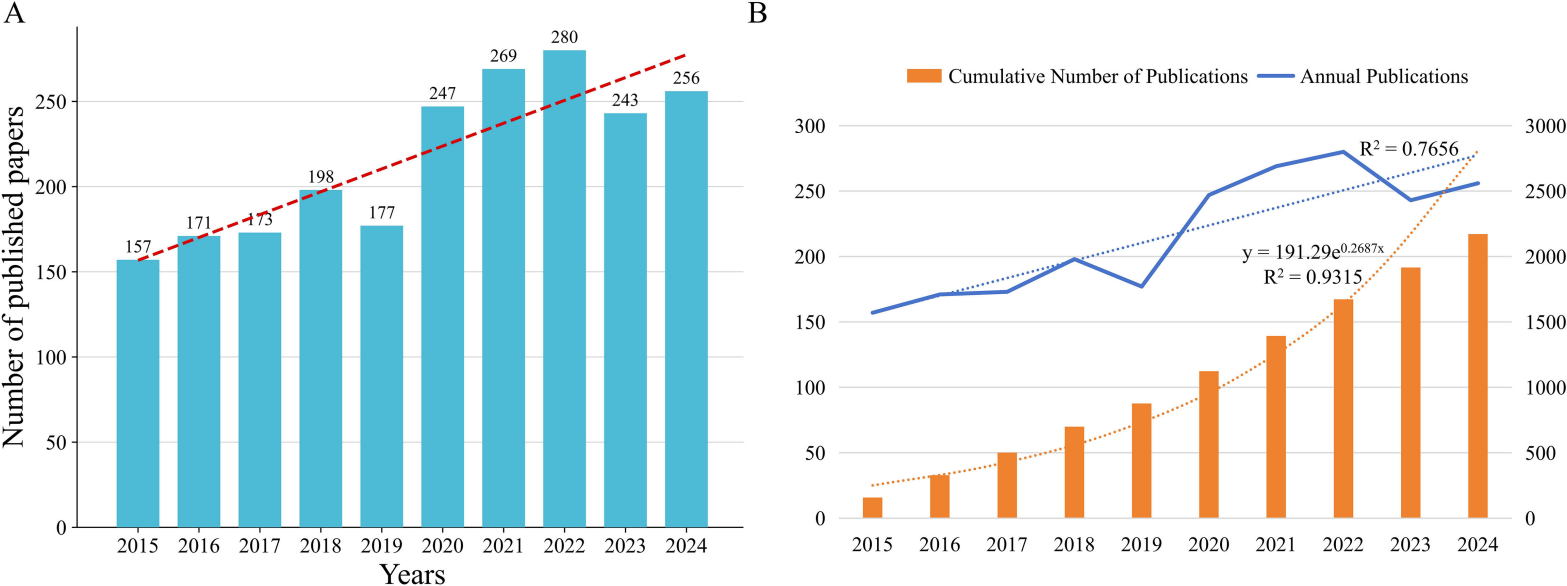
Temporal dynamics of publications on neuroimmune research in lung diseases (2015–2024). **(A)** Annual publication counts. **(B)** Comparison of model fits for cumulative and annual publication volume.

From 2015 through 2024, the annual number of publications addressing neuroimmunology in pulmonary diseases rose each year, reaching a maximum in 2022 with a total of 280 papers. In the subsequent years the publication output underwent only slight fluctuations and showed no marked variation. Over the past decade, cumulative publication totals in this discipline have nearly doubled (**Figure 2A**). To capture the overall change in publication volume more accurately, this study compared the fit between cumulative publication volume and annual publication volume (**Figure 2B**). The fitting curve of cumulative publication volume aligned more closely with actual publication data, with a goodness-of-fit value R²=0.9315, more accurately depicting the long-term trends in this research field and providing more reliable data support for subsequent trend predictions. Overall, these data indicate that research in this field has become a focal point of interest, showing rapid advancements.

### 3.2 National contributions and partnerships

**Figure 3:**
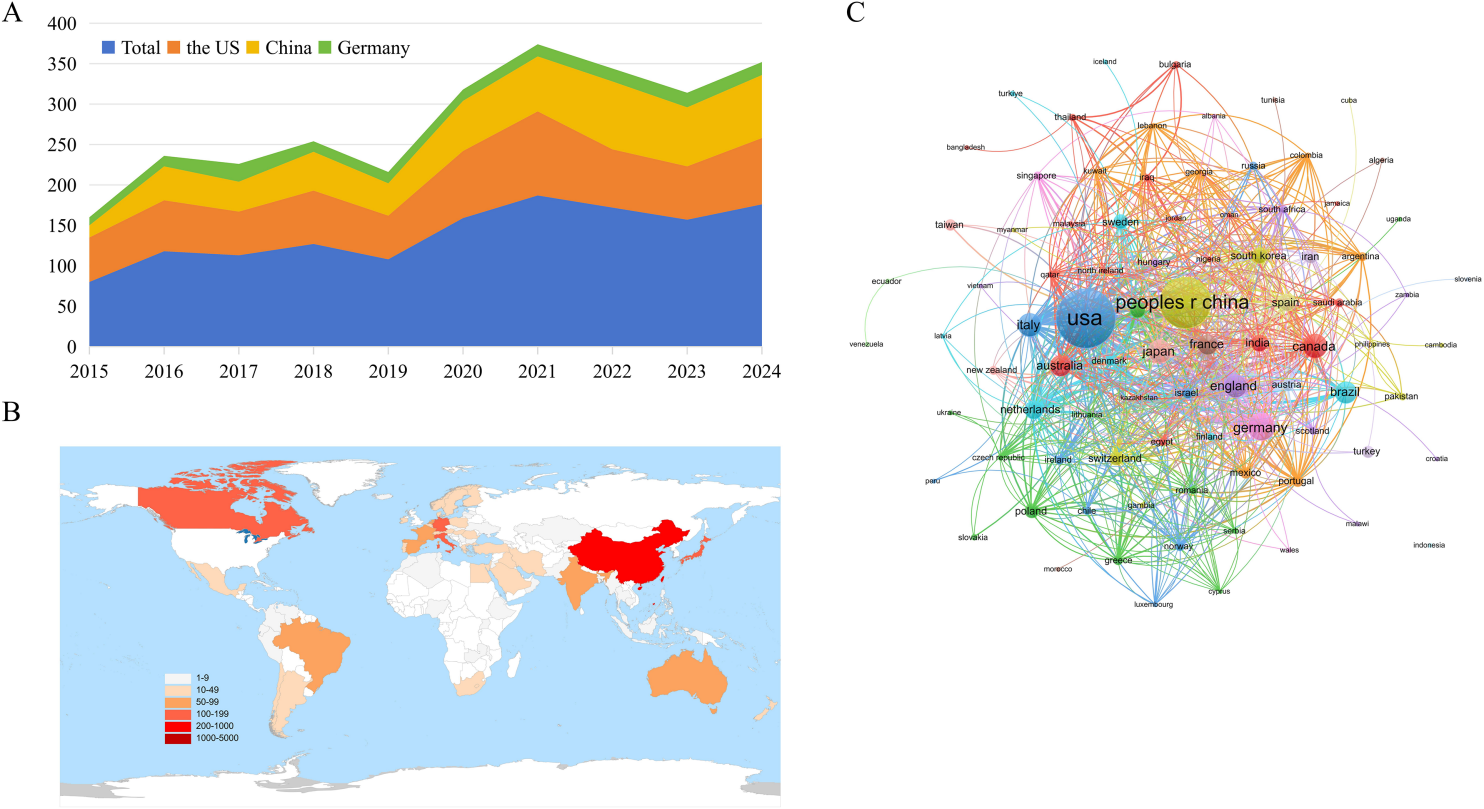
Geographic distribution and international collaboration on the research. **(A)** Top3 contributing countries. **(B)** Global map of outputs: Publications are unevenly distributed geographically, with clear regional concentrations in North America, Europe and East Asia and many countries showing relatively modest output, pointing to opportunities for broader geographic engagement. **(C)** International collaboration network.

We conducted a visual analysis of the publication quantity for each country (**Table 1**). In terms of publication volume, the United States leads with the highest number, followed by China and Germany (**Figure 3A**). Although other countries also contributed, their publication numbers were relatively lower (**Figure 3B**). Specifically, the United States, which leads in publication output, has produced 699 papers and ranks first in both H−index (81) and mean citations per paper (39.12). China ranks second by publication volume (547), with its H−index and average citations slightly below those of the United States. Substantial contributions also come from Germany, the United Kingdom, Italy, and Canada. In VOSviewer, the Attraction and Repulsion parameters were set to 5 and -5, respectively, and all 88 countries were included in the analysis. The prominent role of the United States is additionally reflected in its cross−country collaborations, reinforcing its leadership position (**Figure 3C**). The country−level collaboration network shows that China maintains close partnerships with the Philippines, South Korea, and Switzerland, whereas the United States exhibits strong collaborations with Norway, Italy, and Russia, suggesting that international academic partnerships display regional patterns. Further analysis shows that institutions tend to prefer domestic partners; therefore, we recommend strengthening inter−institutional cooperation at national and international levels to remove barriers that hamper scholarly collaboration.

**Table 1:** Top 10 countries in terms of number of publications.

Rank	Country	NP	NC	H-index	Average per item
1	USA	699	26706	81	39.12
2	China	547	10626	49	20.04
3	Germany	151	4850	38	32.46
4	Canada	114	4132	30	36.71
5	UK	113	4071	38	36.15
6	Italy	112	3855	35	34.57
7	Japan	108	2592	23	24.26
8	Brazil	93	2294	26	25.1
9	Australia	91	2708	27	29.98
10	France	83	2908	31	35.13

### 3.3 Contribution of the issuing institution and journal contribution

**Figure 4:**
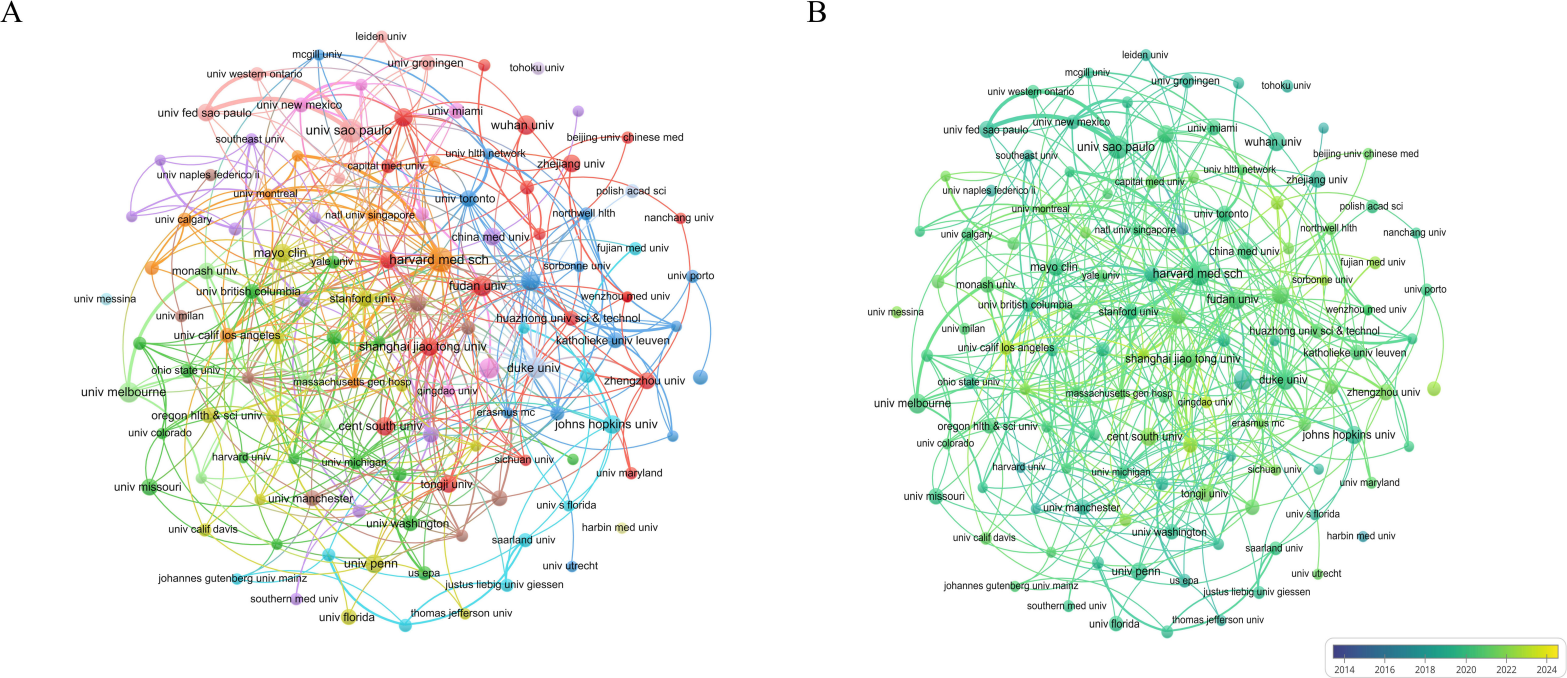
Publication output of top institutions and their collaboration links with leading countries. **(A)** Collaboration network of institutions with ≥8 publications, showing a distinct core-periphery structure. **(B)** Temporal evolution of the collaboration network (color gradient: dark blue-bright green, representing 2015-2024).

**Figure 5:**
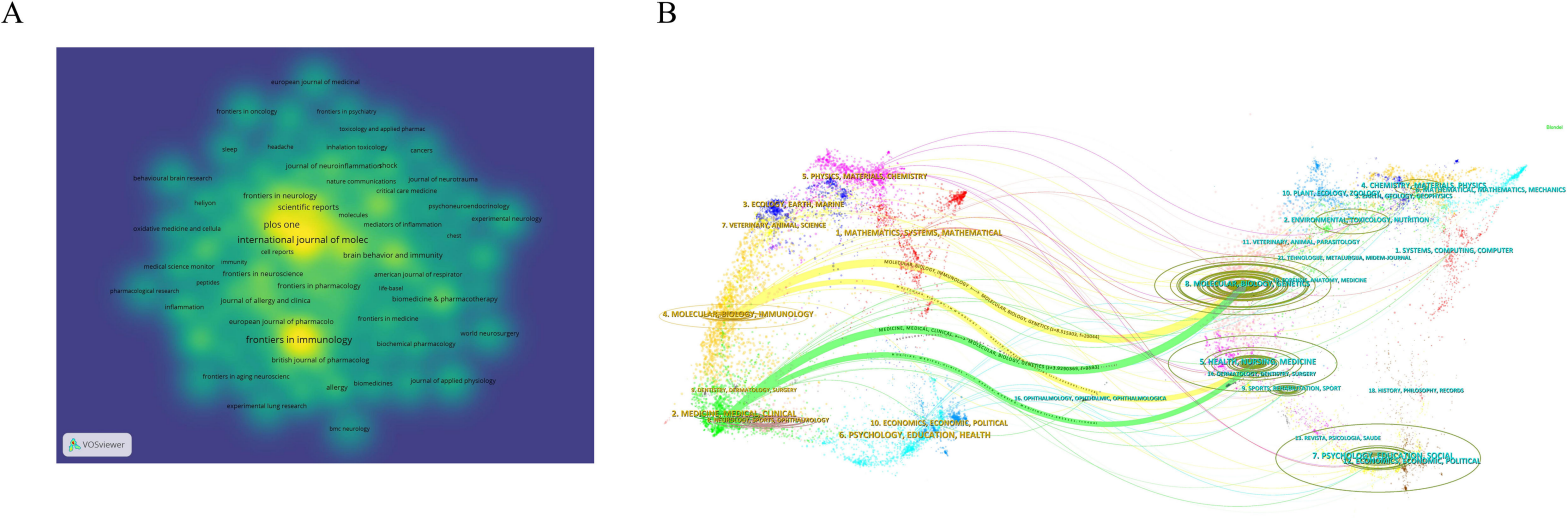
Journal impact and disciplinary knowledge flow **(A)** Journal metrics: Density plot of publication count, citations and IF. **(B)** Dual−map overlay: Major citation paths connect molecular/basic−science journals with clinical and immunology journals, highlighting interdisciplinary exchange.

In bibliometric analyses, major-contributing institutions frequently exhibit strong links with top-contributing countries, and institutional input to the field represents an important bibliometric dimension. In VOSviewer, the minimum number of documents per organization was set to 8, with Attraction = 8 and Repulsion = −2. The publication output of these institutions for 2015–2024 was visualized graphically (**Figure 4A**), The network exhibits a pronounced core-periphery structure, with a rich diversity of node colors reflecting differences in institutional activity and connection strength within the collaboration network. Leading institutions, exemplified by Harvard Medical School (Harvard Med Sch), form a vividly colored core region characterized by dense collaborative links, whereas peripheral institutions are more dispersed and exhibit sparse connections. **Figure 4B** presents the temporal evolution of the collaboration network (color scale from deep blue to bright green representing 2015–2024). The color distribution clearly indicates that the network center is dominated by Harvard Medical School, whose node and surrounding links display the brightest green, signifying the highest recent collaborative activity and continued leadership in global research cooperation. At the same time, Chinese institutions such as Tongji University (Tongji Univ), Fudan University (Fudan Univ), and Shanghai Jiao Tong University (Shanghai Jiao Tong Univ) also appear in vivid green, reflecting a marked increase in their collaborative activity in later years and their emergence as indispensable components of the global collaboration network. **Table 2** presents the top ten institutions by publication count relevant to this analysis. In order to avoid duplicate records and enhance data reliability, publications attributed to “Harvard University,” “Harvard University Medical Affiliates,” and “Harvard Medical School” were merged, deduplicated, and uniformly assigned to “Harvard University.” Final statistics indicate that the University of California System ranked first with 62 publications (Np = 62), followed by Harvard University(Np =58). Harvard University also recorded the largest total citations (NC = 4652) and the highest H−index (31), together reflecting the particularly strong research output quality and influence from the United States. In VOSviewer, the minimum number of documents per source was set to 5, with Attraction = 6 and Repulsion = -4, the density plot in **Figure 5A** illustrates that journal impact is commonly evaluated using publication count, citation frequency, and impact factor (IF). **Table 3** enumerates the ten journals with the greatest publication volume: International Journal of Molecular Sciences and Frontiers in Immunology leads with 56 articles (5.1%), followed by PLOS One with 42 articles (1.9%). Other notable venues include Scientific Reports (31 articles, 1.4%) and Frontiers in Physiology (25 articles, 1.2%). Among the ten, Brain Behavior and Immunity has the highest IF (7.6).

**Table 2:** The top 10 institutions in the number of publication.

Rank	Affiliations	Country	NP	NC	H-index	Average per item
1	University of California System	USA	62	2425	23	39.4
2	Harvard University	USA	58	4652	31	81.03
3	Institut National de la Sante et de la Recherche Medicale (Inserm)	France	50	1627	25	32.6
4	University system of Ohio	USA	38	1511	19	39.95
5	Universidade De Sao Paulo	Brazil	34	660	16	20.24
6	Centre National De La Recherche Scientifique	France	30	872	18	29.13
7	Imperial College London	UK	30	1040	17	34.83
8	National Institutes of Health NIH USA	USA	30	975	15	32.63
9	Pennsylvania Commonwealth System of Higher Education	USA	30	830	16	27.73
10	State University System of Florida	USA	30	736	16	25

**Table 3:** Top 10 sources by volume of published literature.

Rank	Journal	NP	NC	IF(2024)	H-index	Average per item
1	International Journal of Molecular Sciences	56	1298	4.9	20	23.46
2	Frontiers in Immunology	56	1280	5.9	20	22.95
3	PLOS One	42	767	2.6	17	18.31
4	Scientific Reports	31	476	3.9	14	15.35
5	Frontiers in Physiology	25	360	3.4	12	14.52
6	International Immunopharmacology	23	471	4.7	15	20.52
7	Brain Behavior and Immunity	21	1110	7.6	13	52.9
8	American Journal of Physiology Lung Cellular and Molecular Physiology	19	381	4.3	11	20.21
9	Frontiers in Neurology	19	403	2.8	9	21.74
10	Frontiers in Neuroscience	16	376	3.2	11	23.63

Journal influence is commonly reflected by how frequently its articles are cited, which indicates its prominence within a research area. **Table 4** presents the top ten co−cited journals; five exceed 1,800 citations. Nature ranks first with 2,592 citations, followed by PLOS One (2,529), Proceedings of the National Academy of Sciences of the USA (2,243), American Journal of Respiratory and Critical Care Medicine (2,185) and Journal of Immunology (1,874). Notably, the New England Journal of Medicine ranked seven, with a very high impact factor (IF = 78.5). Journals of this caliber serve as central channels for the rapid dissemination of novel findings in this area.

**Table 4:** The top 10 journals by co-citation.

Rank	Cited journal	Co-citation	IF (2024)	Quartile in category
1	Nature	2592	48.5	Q1
2	PLOS One	2529	2.6	Q2
3	Proceedings of the National Academy of Sciences of the USA	2243	9.1	Q1
4	American Journal of Respiratory and Critical Care Medicine	2185	19.4	Q1
5	Journal of Immunology	1874	3.4	Q2
6	Journal of Allergy and Clinical Immunology	1732	11.2	Q1
7	New England Journal of Medicine	1456	78.5	Q1
8	Cell	1334	42.5	Q1
9	Journal of Neuroscience	1334	4	Q1
10	American Journal of Physiology Lung Cellular and Molecular Physiology	1321	3.5	Q1

A dual−map overlay visualizes the thematic distribution of the literature (**Figure 5B**), illustrating the subject distribution of academic journals, citation trajectories, and the evolution of research centers. This visualization helps in understanding how various disciplines interact, the impact of citations across different fields, and the shifts in research focus over time (20). The colored lines represent the connections between citations, with the left side indicating the citing domains and the right side showing the cited domains. Based on the displayed results, we identified four major colored citation paths. Works from molecular, biological, and immunology disciplines are predominantly cited by literature in molecular biology, genetics, and health/nursing/medical fields. Conversely, papers originating from medical, clinical, and healthcare domains receive most citations from those same molecular, biological, genetics, and health/nursing/medical areas.

It is evident that the application of neuroimmunology in pulmonary diseases spans several fields, including molecular, biological, genetic, immunological, health, nursing, and medical studies.

### 3.4 Analysis of authors

Identifying influential authors in a specific field through their foundational works provides a pathway to understanding classical theories. **Table 5** lists the top 10 most productive authors. They have published a total of 100 publications, accounting for 4.6% of all submitted articles. Fryer, Allison D (11 publications) from Oregon Health & Science University ranked first., Tiberio, IFLC (11 publications) from the Universidade de São Paulo followed closely. Prado, Carla (11 publications) and Prado, Marco (10 publications) are both from Universidade de São Paulo. Isaac M. Chiu (10 publications) from Harvard University and Jacoby, David B (10 publications) from Oregon Health & Science University also appear among the top contributors. Isaac M. Chiu (USA) has the highest H−index and the highest average citations per item, indicating his work has attracted particularly broad attention. Overall, most of the top ten authors are based in the United States and Brazil, reflecting a prominent cohort of neuroimmunology researchers in these countries.

**Table 5:** Top 10 most influential researchers in the field based on number of publications.

Rank	Author	NP	NC	Country	Affiliations	H-index	Average per item
1	Fryer, Allison D	11	315	USA	Oregon Health Science University	8	30
2	Tibério, IFLC	11	185	Brazil	Universidade De Sao Paulo	6	19.18
3	Prado, Carla	11	167	Brazil	Universidade De Sao Paulo	6	17.55
4	Prado, Marco	10	170	Brazil	Universidade De Sao Paulo	6	19.2
5	Isaac M Chiu	10	2108	USA	Harvard University	10	212.8
6	Jacoby, David B	10	266	USA	Oregon Health Science University	8	28.1
7	Prado, Vania Ferreira	10	170	Brazil	Universidade De Sao Paulo	6	19.2
8	Campen, Matthew J	9	372	USA	University of New Mexico	7	43
9	Penninx, BWJH	9	297	Netherlands	Vrije Universiteit Amsterdam	8	34.22
10	Xu, Fadi	9	74	USA	Lovelace Respiratory Research Institute	6	9.22

### 3.5 Visualization and cluster analysis of co-cited references

**Figure 6:**
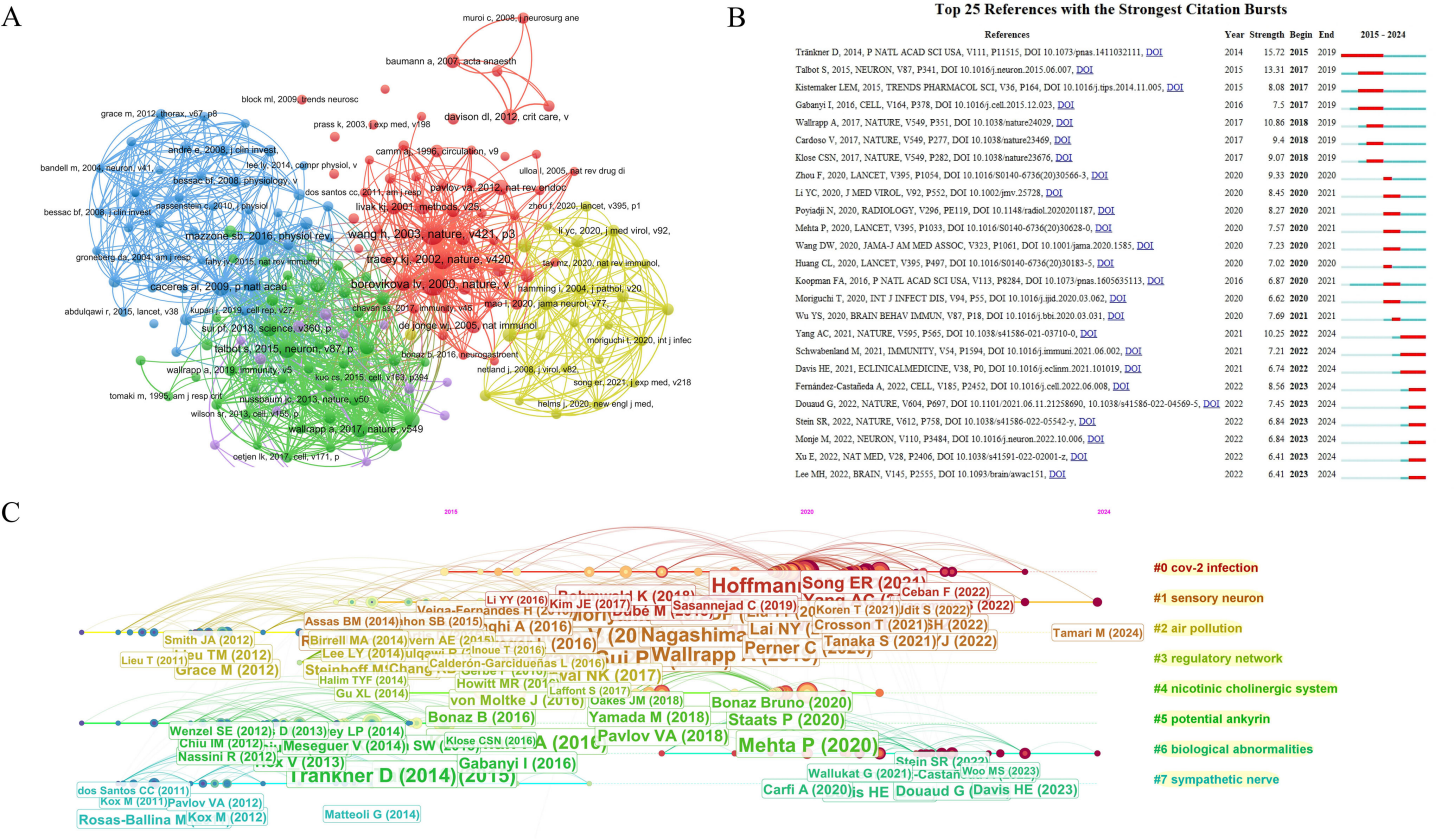
Co−citation structure and temporal citation bursts. **(A)** Co−citation network of references (180 of 129,933 cited works selected). Links indicate co−citations, node colors denote thematic clusters. **(B)** Top 25 references with the strongest citation bursts. **(C)** Citation bursts (length, strength, timing); the top eight burst references concern regulatory network, CoV−2 infection, potential ankyrin, nicotinic cholinergic system, air pollution, sensory neuron, biological abnormalities and sympathetic nerve.

In contrast to broad citation analyses, co−citation networks highlight topic clusters that are tightly linked to particular disciplines. Co−cited works are those jointly referenced by subsequent authors and are commonly regarded as constituting the foundational knowledge of a given domain. This approach helps to identify key publications that significantly contribute to the development of knowledge within the field, showcasing the interconnections between different works and highlighting fundamental theories and methodologies that shape the area of study. (21) From the large pool of references, 180 of the 129,933 cited works in the retrieved publications were selected for co−citation analysis. The minimum number of citations per cited reference was set to 17, with Attraction = 6 and Repulsion = -2. Lines connecting two nodes indicate that the two references co−occurred in the same article; shorter links denote a stronger relationship between the two publications. Node color represents cluster membership. (**Figure 6A**) Cluster 1 (red): 56 references, published in high−impact journals such as Nature and Immunity, focusing on neuro-immune interactions, with a particular emphasis on the regulatory mechanisms of the vagus nerve-mediated cholinergic anti-inflammatory pathway in pulmonary inflammatory diseases such as sepsis and acute respiratory distress syndrome (ARDS). It provides a crucial theoretical basis for understanding how the nervous system actively regulates pulmonary immune responses, thereby influencing the onset and progression of lung diseases. Cluster 2 (green): 44 references, the core research direction of this cluster focuses on the interactions between sensory neurons and pulmonary immunity. It specifically investigates the mechanisms by which nociceptors regulate the function of immune cells—such as neutrophils and group 2 innate lymphoid cells (ILC2s)—through the release of neuropeptides in pulmonary diseases like bacterial pneumonia and asthma. Meanwhile, it reveals the critical role of pulmonary neuroendocrine cells (PNECs) as airway sensors in environmental stimulus perception and local immune regulation. This provides a vital theoretical framework for a deeper understanding of the role of the neuro-immune regulatory network in the pathogenesis and progression of lung diseases. Cluster 3 (blue): 42 references, centered on the interplay between airway sensory nerves and pulmonary immunity. Investigations focus on how airway nociceptors (e.g., TRPV1+ neurons) detect environmental stimuli, pathogen-associated molecular patterns (PAMPs), and inflammatory mediators. Subsequently, they regulate the function of immune cells—including airway epithelial cells, mast cells, macrophages, and T cells—through the release of neuropeptides such as calcitonin gene-related peptide (CGRP) and Substance P. Cluster 4 (yellow): 25 references, temporally concentrated in 2020-2021, this research cluster emerged in close parallel with the outbreak of the COVID-19 pandemic, describing the impact of viral infections, particularly COVID-19, on the nervous system and the underlying neuro-immune mechanisms. It specifically investigates the direct invasion and injury of the central nervous system by viruses such as SARS-CoV-2. Furthermore, this cluster delves into the associations between post-viral neuroinflammation, immune cell infiltration, and cytokine storms, as well as potential therapeutic targets. This research provides critical data for understanding the neuropathological mechanisms of viral infections and represents a rapid response and cutting-edge exploration within the field of neuroimmunology concerning the interplay between viral infections and the nervous system amidst a global public health crisis. Cluster 5 (purple): 13 references, converging on the regulatory role of the neuro-immune-metabolic axis in pulmonary diseases and systemic inflammation. It elucidates the mechanisms by which the nervous system influences immune cell metabolic reprogramming and the progression of pulmonary fibrosis through the modulation of metabolic pathways—including glycolysis and lipid metabolism—and metabolic sensors such as AMPK and mTOR. **Figure 6B** displays the 25 references with the most pronounced citation bursts. The study by Dimitri Tränkner et al. exhibited the highest burst strength (15.72); it highlights a key regulatory role of TRPV1−expressing sensory neurons in vagal ganglia in airway hyperresponsiveness that can act independently of immune−inflammatory components, suggesting novel neural−targeted therapeutic approaches for asthma (22). **Figure 6C** illustrates representative references with respect to burst duration, strength, and timing. The generated network comprised 1,442 nodes and 3,584 links, with modularity Q = 0.8764 and silhouette S = 0.9571, indicating that the clustering solution is both significant and reliable. The top eight recurring co−citation topics included the “regulatory network,” “CoV−2 infection,” “nicotinic cholinergic system,” “air pollution,” “sensory neuron,” “potential ankyrin,” “biological abnormalities, “and “sympathetic nerve.”.

### 3.6 Analysis of highly cited articles

**Figure 7:**
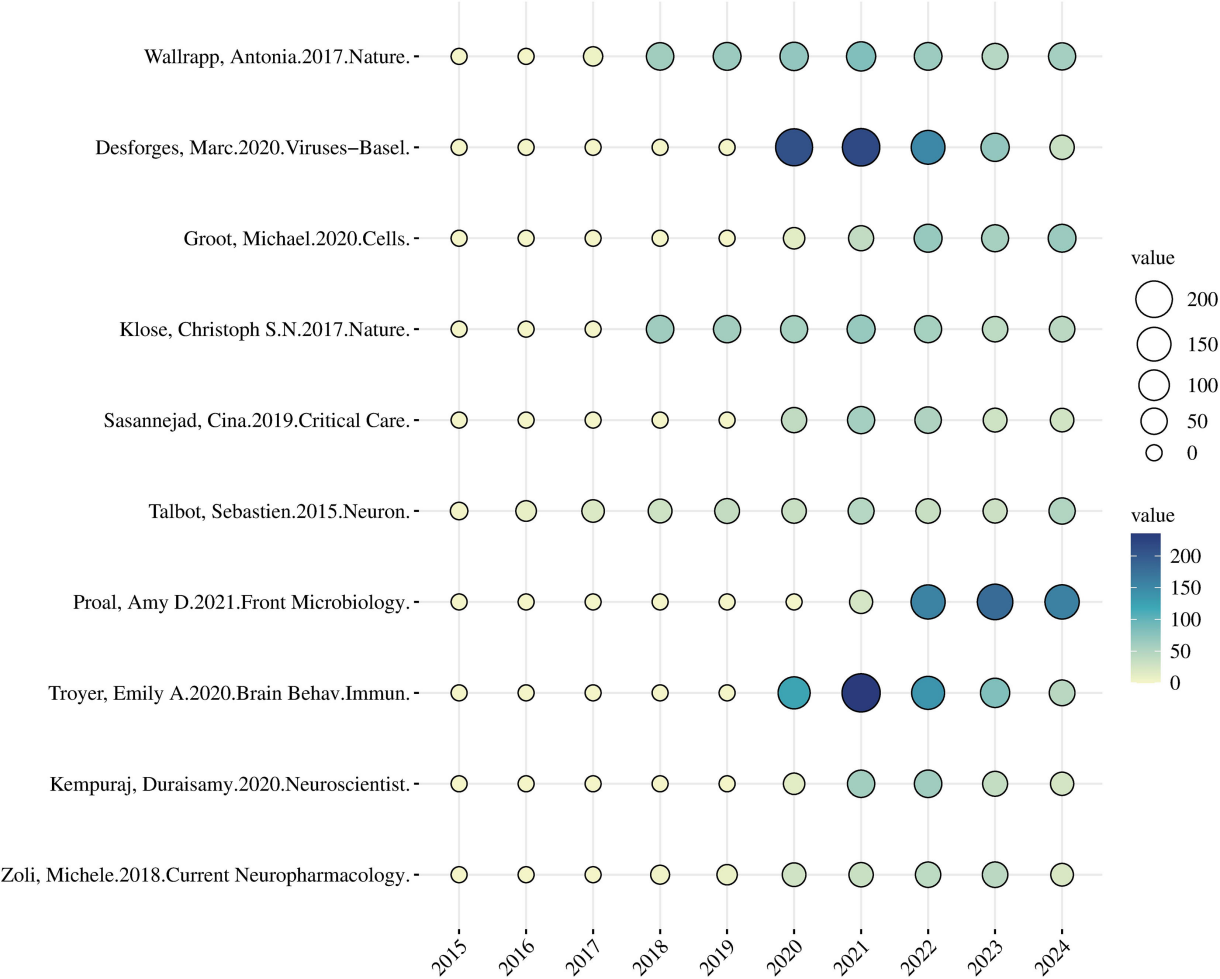
Ranking and visualization of highly cited articles. Node size and depth is proportional to total citations, highlighting the research core and major knowledge hubs.

**Table 6** presents articles ranked in descending order by total citation counts. Consistent with **Figure 7**, article nodes with larger citation numbers are depicted as bigger and mark the research core. The ten most−frequently cited articles were mainly published from 2017 through 2020. Notably, four studies each accumulated more than 400 citations. One seminal paper, published in Viruses-Basel (IF = 3.5), was cited 684 times (23). This study reveals that respiratory viruses (with a focus on human coronaviruses) can breach the respiratory barrier and invade the central nervous system through hematogenous or neural retrograde pathways. The resulting neuroimmune dysregulation not only mediates neurological pathologies such as encephalitis and demyelination but also modulates systemic immune states via virus-host immune interactions. These findings provide critical theoretical support for elucidating the regulatory role of the neuroimmune network in respiratory virus-induced pulmonary diseases and the immune mechanisms linking pulmonary and neurological pathologies. Furthermore, they lay an important foundation for advancing research on neuroimmune-targeted interventions in pulmonary disease treatment. Subsequently, another highly cited article in Brain Behavior and Immunity (IF = 7.6) received 625 citations (24). In addition, two core papers published in Nature (IF = 48.5) jointly revealed that the neuropeptide NMU–NMUR1 axis regulates type 2 innate lymphoid cells (ILC2s) in the lung, identifying this pathway as a key mechanism of pulmonary inflammation and providing a mechanistic and interventional basis for neuroimmune regulation of lung disease; these two papers together received 853 citations (25, 26).Together, these ten highly cited publications have constructed the intellectual framework of neuroimmunology in pulmonary disease: they clarify how neuropeptides modulate pulmonary immune responses, document viral neuroinvasion of the respiratory tract, describe vagal−mediated brain–gut/brain–lung immune regulation, and define COVID−19 neuropathological associations. Collectively, these studies form a multi−layered body of evidence—from molecular neuroimmune mechanisms to cross−tissue regulatory networks and clinical disease correlations—providing integrated basic−to−clinical support for the role of neuroimmune interactions in lung disease.

**Table 6:** The top 10 highest cited articles.

Rank	Year	Article	IF	Total citation	Type of study
1	2020	Human Coronaviruses and Other Respiratory Viruses: Underestimated Opportunistic Pathogens of the Central Nervous System? (PMID: 31861926)	3.5	684	review
2	2018	Are we facing a crashing wave of neuropsychiatric sequelae of COVID-19? Neuropsychiatric symptoms and potential immunologic mechanisms (PMID: 32298803)	7.6	625	review
3	2021	Long COVID or Post-acute Sequelae of COVID-19 (PASC): An Overview of Biological Factors That May Contribute to Persistent Symptoms (PMID: 34248921)	4.5	522	review
4	2017	The neuropeptide NMU amplifies ILC2-driven allergic lung inflammation (PMID: 28902842)	48.5	459	article
5	2017	The neuropeptide neuromedin U stimulates innate lymphoid cells and type 2 inflammation (PMID: 28869965)	48.5	394	article
6	2015	Silencing Nociceptor Neurons Reduces Allergic Airway Inflammation (PMID: 26119026)	15.3	292	article
7	2020	Sorting Mechanisms for MicroRNAs into Extracellular Vesicles and Their Associated Diseases (PMID: 32331346)	5.2	244	review
8	2019	Long-term cognitive impairment after acute respiratory distress syndrome: a review of clinical impact and pathophysiological mechanisms (PMID: 31718695)	9.3	207	article
9	2020	COVID-19, Mast Cells, Cytokine Storm, Psychological Stress, and Neuroinflammation (PMID: 32684080)	3.9	201	article
10	2018	Neuronal and Extraneuronal Nicotinic Acetylcholine Receptors (PMID: 28901280)	5.3	180	review

### 3.7 Analysis of hotspots in research

**Figure 8:**
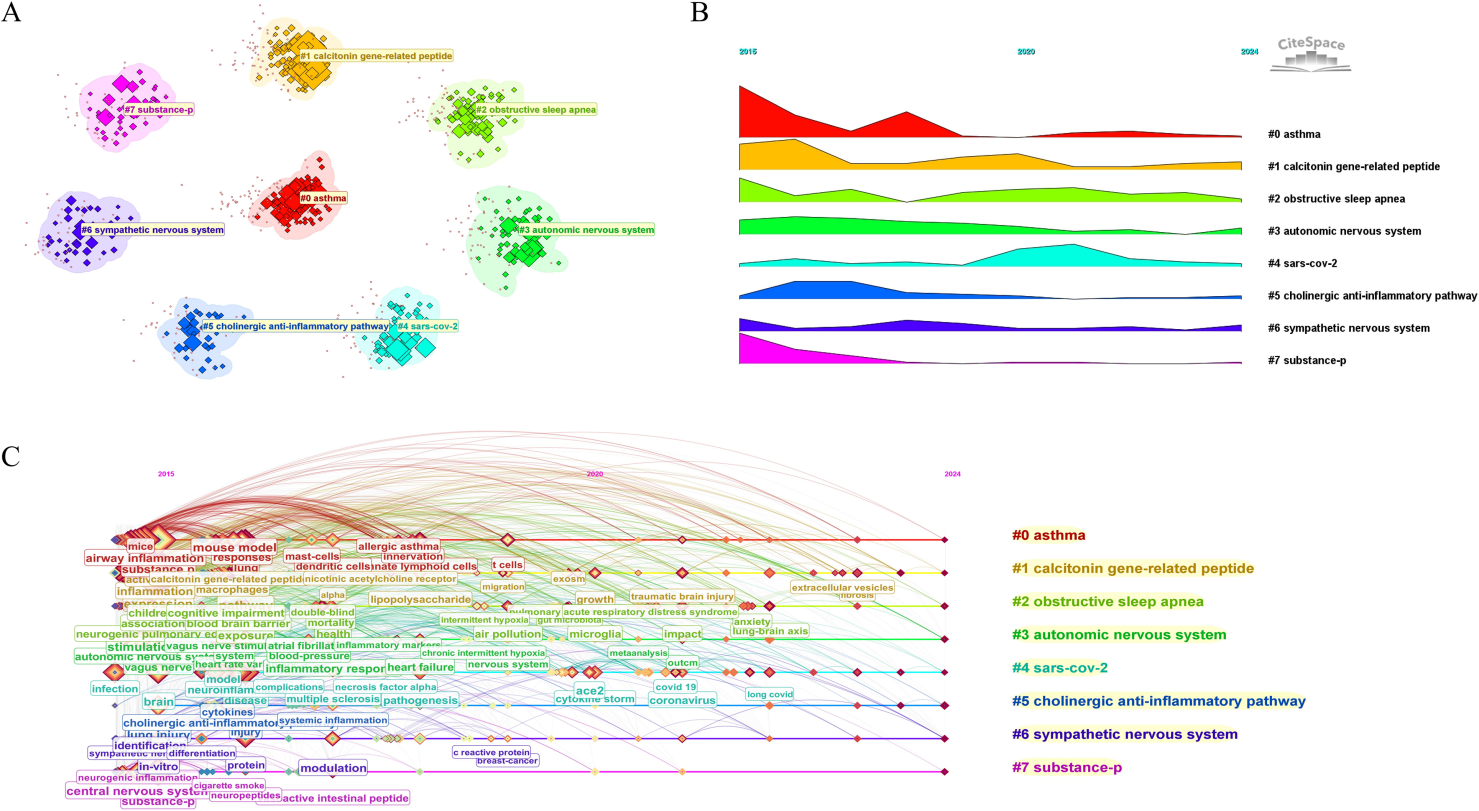
Keyword clustering and temporal evolution of research hotspots. **(A)** Keyword−cluster visualizatio81n by LLR algorithm. LLR, log−likelihood ratio. **(B)** Temporal evolution of research clusters (2015-2024), strip height indicates cluster activity. **(C)** Persistent hotspots and their interrelationships.

The keyword co-occurrence network, generated using predefined parameters and the VOSviewer clustering algorithm, comprised 434 nodes and 3,023 links, with a modularity Q value of 0.3473, indicating a moderately structured thematic organization. The clustered keyword map is visualized in **Figures 8A**, while **Figure 8B** illustrates the temporal evolution and distribution of research hotspots across the field from 2015 to 2024. Each colored bar represents a distinct research cluster; the height of a bar reflects the relative prominence of that theme over time. This comprehensive analysis reveals the dynamic shifts and structural organization of research priorities in neuroimmunology related to pulmonary diseases over the past decade. Core themes revolve around neuroimmune interactions, extending to specific respiratory conditions—including asthma and obstructive sleep apnea and encompassing key regulatory mechanisms such as calcitonin gene-related peptide (CGRP) signaling and autonomic nervous system (ANS) modulation. Notably, SARS−CoV−2–related studies surged sharply around 2020, forming a pronounced peak of scholarly attention, highlighting emerging trends toward immune regulation, respiratory comorbidities, multimorbidity, and interdisciplinary integrations. The top eight clusters identified are: #0 asthma, #1 calcitonin gene-related peptide, #2 obstructive sleep apnea, #3 autonomic nervous system, #4 sars-cov-2, #5 cholinergic anti-inflammatory pathway, #6 sympathetic nervous system and #7 subtance-p. These topics have consistently remained central to the field, as further substantiated by their sustained presence and high linkage density in the network. (**Figure 8C.**).

### 3.8 Cross-database validation with PubMed

To assess the robustness of our primary findings and mitigate potential biases inherent to a single database, we performed a cross-validation using an independent dataset retrieved from PubMed. Although the initial search result from PubMed (1,970 publications after screening) differed in volume from the WoS dataset (2,171 publications after screening), reflecting their fundamental differences in scope—where WoS offers broader coverage of interdisciplinary, basic science, and high-impact international journals, while PubMed maintains a more concentrated focus on clinical and biomedical literature, including numerous regional clinical journals and earlier-stage research outputs—a comparative analysis of key bibliometric indicators revealed a high degree of consistency.

**Figure 9:**
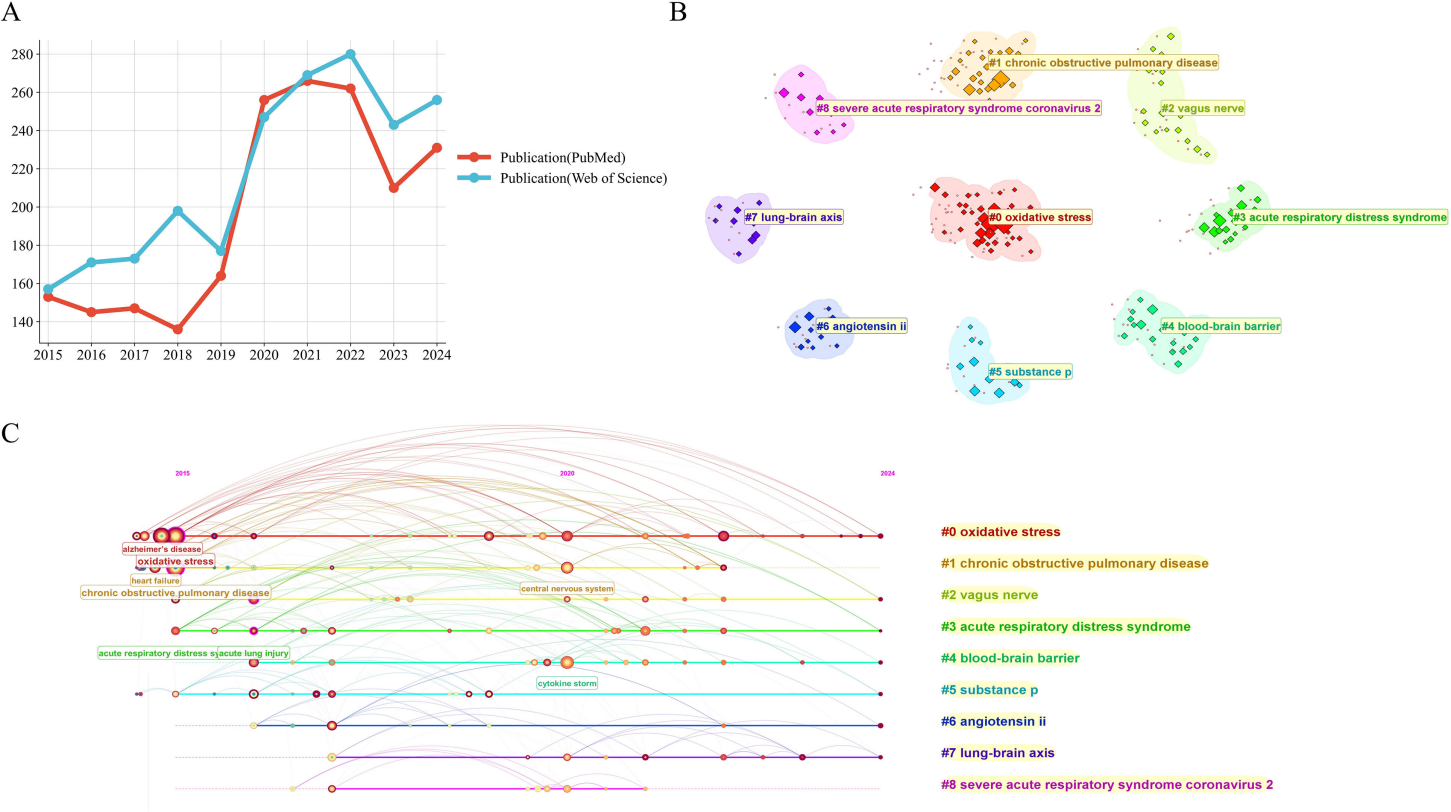
Cross-database validation of research trends and thematic focus **(A)** Annual publication trends in WoS and PubMed (2015–2024). **(B)** Keyword clustering analysis based on the PubMed dataset, demonstrating the main research topics in this field. Each cluster is represented by a distinct color and labeled with its most representative keyword. **(C)** Keyword clustering timeline map. Each colored line represents a clustered research theme, with its horizontal extension from left to right indicating the temporal span of the theme. The size of the nodes reflects the frequency of keyword occurrence.

We compared the annual publication trends between the two databases (**Figure 9A**). The data from WoS and PubMed clearly delineate parallel growth trajectories. Both datasets show a substantial increase in publication output over the past decade, peaking around 2021-2022. The strong alignment of these trends robustly confirms that the expansion of research activity in pulmonary neuroimmunology is a genuine and reproducible phenomenon, not an artifact of a single database’s coverage.

A comparative keyword analysis revealed both shared foundations and complementary perspectives between WoS and PubMed in the neuroimmunity-pulmonary disease field (**Figure 8A**, **9B**, **8C**, **9C**). Both databases emphasize core topics such as neuroimmunity and pulmonary diseases (e.g., asthma, chronic obstructive pulmonary disease), and each registers parallel interest in SARS−CoV−2-related research. Distinct patterns are evident: WoS is comparatively enriched for mechanistic concepts (e.g., calcitonin gene−related peptide/CGRP, autonomic nervous system), whereas PubMed preferentially highlights clinically oriented disease entities (e.g., acute respiratory distress syndrome) and pathophysiological processes (e.g., oxidative stress, cytokine−driven systemic inflammation). These differences are consistent with the intrinsic scopes of the databases: WoS more broadly covers interdisciplinary basic−science dimensions, while PubMed more closely reflects clinical and practice−driven concerns.

## 4 Discussion

This is the first bibliometric study to map neuroimmunology’s role in pulmonary diseases. We queried the Web of Science Core Collection (SCI−Expanded) and applied VOSviewer and CiteSpace to analyze publication trends and research hotspots in pulmonary neuroimmunology. A total of 2,171 articles and reviews published between 2015 and 2024 were retrieved. Although annual outputs fluctuated modestly during the decade, the overall trajectory shows an increase in publications, suggesting growing research interest in this area.

Publications are globally dispersed, although regional output demonstrates marked heterogeneity. The geographic distribution of these studies is shown in **Figure 3B**. The United States leads with 699 publications, followed by China (547), Germany (151), and Canada (114). The U.S. ranks first among the top ten countries/regions by publication count (Np), highlighting its high productivity in this area. Furthermore, seven of the top ten contributing institutions are located in the United States, reflecting a concentration of leading centers. These institutional strengths help explain the substantial influence the United States has exerted in this discipline over the past decade.

Moreover, compared with China, the United States shows a relatively higher H−index and Nc (total citations). This can be attributed to the discoveries and research by American researchers regarding the “parasympathetic anti-inflammatory pathway”. Borovikova et al. first confirmed in 2000 that vagus nerve stimulation could inhibit macrophage release of pro-inflammatory factors through acetylcholine. This pivotal finding has significantly influenced subsequent studies in the field, underscoring the importance of neuroimmunological interactions in regulating inflammation (27). Subsequently, in 2003 Wang and colleagues clarified the pivotal contribution of the α7 nicotinic acetylcholine receptor to this pathway. Their work offered more detailed understanding of how this receptor conveys parasympathetic anti−inflammatory signaling, emphasizing its relevance to neuroimmune regulation and its prospective therapeutic value for inflammatory conditions (28). This comprehensive elucidation of the physiological phenomenon to molecular mechanisms not only established a theoretical foundation for neuroimmunological regulation but also provided a clear direction for subsequent research. It guided researchers worldwide to conduct in-depth studies across various disease models, positioning the United States as a hub for knowledge exchange and research collaboration in this field. As a result, the U.S. demonstrates outstanding academic influence and a core position in bibliometric analyses. It is recommended that scholars and affiliated institutions in this discipline in China improve the quality of their publications. Similarly, in Brazil and Japan, there are also discrepancies in both the quantity and quality of publications, indicating the need for enhanced research efforts and academic output in the field of neuroimmunology.

Regarding institutional affiliations, six of the top ten organizations are based in the United States. Harvard University stood out with 58 publications, 4,652 total citations, and an H−index of 31; its mean citations per article reached 81.03, reflecting both the high quality of its outputs and its leading global position in this field. Other top US institutions, including the University of California system and the University system of Ohio, also contributed high volumes of publications and influential findings, together forming a clustered national strength in this research area. Although institutions from France and the United Kingdom are fewer in number, both the French National Institute of Health and Medical Research (INSERM) (29) and Imperial College London (30) have demonstrated significant research strength. INSERM ranks high in publication volume, while Imperial College London exhibits a commendable average citation per publication, reflecting their impactful contributions to the field of neuroimmunology. At the researcher level, the most prolific scholar in this field is Allison D. Fryer from Oregon Health & Science University, USA, followed by IFL C. Tibério and Carla Prado from the Universidade de São Paulo, Brazil. Notably, although Isaac M. Chiu from Harvard University ranks fifth in publication volume, he holds the highest total citation count (2,108 times) and the highest average citations per paper (212.8 times) among all scholars, with an H-index of 10, underscoring the exceptional impact of his research. Furthermore, multiple members of the Prado research team at the Universidade de São Paulo (Carla Prado, Marco Prado, and Vania Ferreira) are listed within the top 10, indicating that this institution has established a stable and highly productive research pipeline in the field of neuroimmunity and pulmonary diseases. This reflects sustained institutional investment and systematic team development in this area. Such a distribution suggests that research in neuroimmunity and pulmonary diseases is characterized by a healthy, multi-center, and multi-national collaborative landscape, featuring both high-impact scientists like Isaac M. Chiu and several well-structured, consistently productive research teams. Future research efforts should focus on the work of these leading scholars while also tracking the progress of highly productive teams, such as the one at the Universidade de São Paulo, to comprehensively grasp the frontier directions and developmental trends in this field. This diversified collaborative model lays a solid foundation for continued innovation and breakthrough advancements in the discipline. Based on the journal analysis, this study reveals that knowledge dissemination in the field of neuroimmunology and pulmonary disease exhibits distinct interdisciplinary characteristics. The top ten journals comprise a diversified knowledge system, incorporating both widely influential general journals and authoritative journals from various specialized fields. This diverse composition underscores the multifaceted nature of research within this area, allowing for cross-disciplinary collaboration and the integration of diverse perspectives, which can enhance the understanding and treatment of neuroimmunological and pulmonary diseases.

Journals such as The New England Journal of Medicine, Cell, and Nature primarily publish landmark breakthrough studies. Notably, research on immune checkpoint inhibitor (ICI)-associated pneumonia has become a significant knowledge foundation in this field. Recent advancements, such as single-cell RNA sequencing of bronchoalveolar lavage fluid and the identification of biomarkers (including autoantibodies), are enhancing our understanding of ICI-related pneumonia. These developments contribute to deeper insights into the mechanisms underlying these conditions and may lead to improved diagnostic and therapeutic approaches (31). At the same time, Nature published an authoritative review (32). This study provides an in-depth elaboration on the molecular mechanisms by which the vagus nerve regulates pulmonary immune responses, establishing an important theoretical framework for this field.

**Figure 10:**
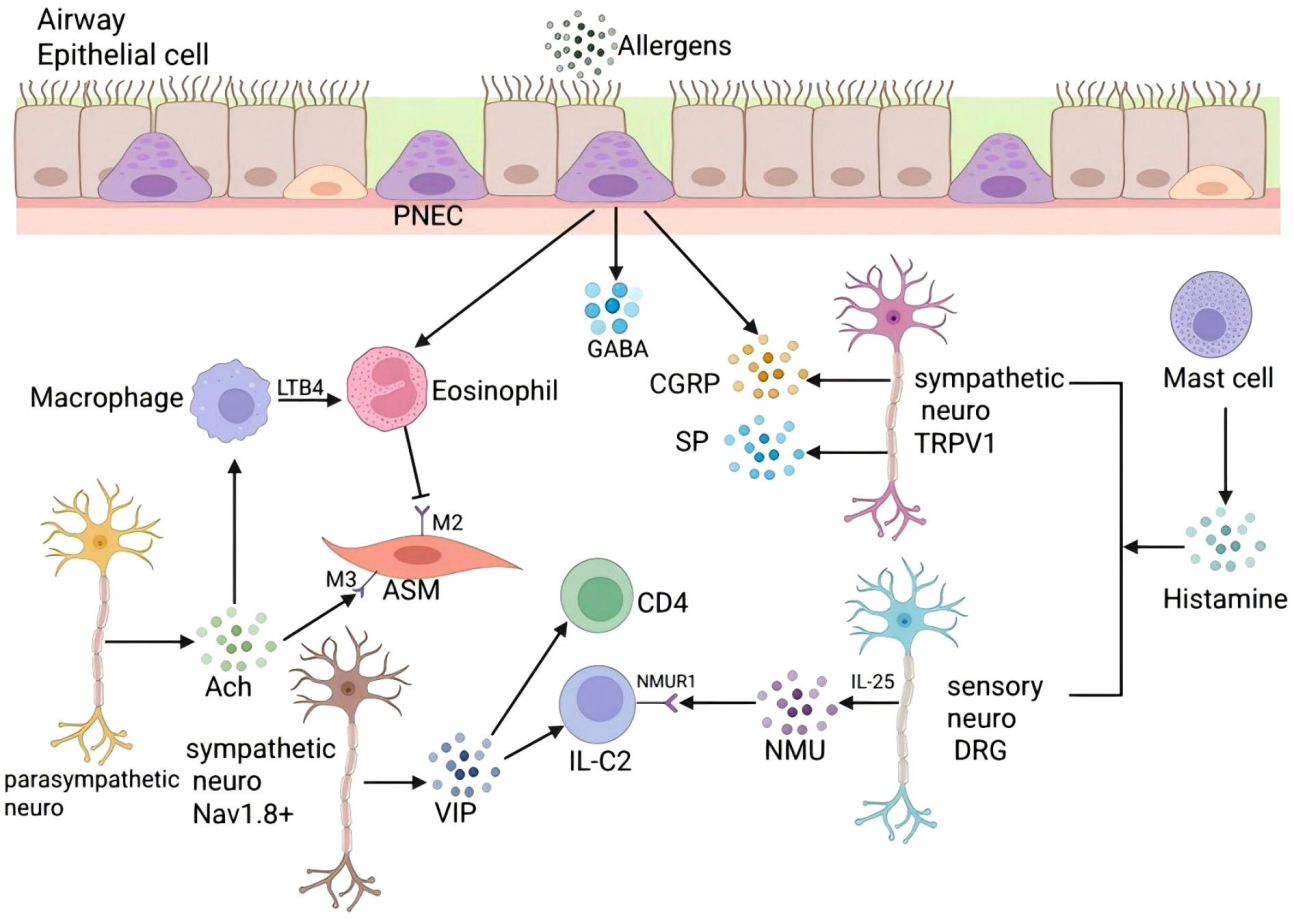
Schematic of neuroimmune mechanisms contributing to allergic asthma. Pulmonary neuroendocrine cells (PNEC) express high levels of CGRP and release GABA in response to eosinophil extracellular traps (EETs), amplifying inflammation. TRPV1+ vagal/sympathetic fibers release neuropeptides such as CGRP and substance P **(SP)**, promoting leukocyte recruitment and cytokine secretion. Nav1.8+ pulmonary nociceptors, when stimulated by IL−5, secrete vasoactive intestinal peptide (VIP), which activates CD4+ T cells and group 2 innate lymphoid cells (ILC2), creating a positive feedback loop that worsens airway inflammation; blockade of sodium channels attenuates this effect. IL−25 stimulates dorsal root ganglia (DRG) sensory neurons to release neuromedin U (NMU), which acts via NMUR1 on ILC2 to potentiate type 2 allergic responses. Acetylcholine (ACh) from parasympathetic neurons acts on airway epithelial and immune cells through muscarinic and nicotinic receptors to induce bronchoconstriction and enhance inflammatory signaling. Solid arrows indicate established activating pathways; dashed arrows indicate feedback loops or indirect effects.

The role of professional journals is reflected in the construction of a systematic body of specialized knowledge. Nature, as the most co-cited journal, represent frontier breakthroughs at the intersection of neuroimmunology and pulmonary disease. Collectively, they advance our understanding of the lung-brain axis and neuroimmune interaction mechanisms from multiple complementary angles, and they provide critical mechanistic evidence for the pathogenesis of pulmonary inflammatory disorders such as allergic asthma. Rather than remaining limited to descriptive clinical observations or isolated immune responses, these investigations systematically delineate how neuropeptides, sensory neural pathways, and the pulmonary microbiome function as pivotal nodes that couple local lung pathology with systemic immune responses and central nervous system activity. These findings demonstrate that the lung is not only a respiratory organ but also a major target and mediator in neuroimmune disease processes, with neuroimmune crosstalk playing a central role in diverse chronic airway diseases. Here, we focus on recent research regarding allergic asthma. Allergic asthma is one of the most prevalent chronic inflammatory diseases in the world (33). In asthma pathogenesis, crosstalk between nociceptive neurons and immune cells is pivotal. For example, activation of TRPV1+ vagal fibers may increase airway hyperresponsiveness, partly through release of neuropeptides (e.g., CGRP and substance P) that drive leukocyte recruitment and cytokine release (22, 34). Research on pulmonary nociceptors, particularly Nav1.8+ neurons, indicates that under IL-5 stimulation, these neurons produce vasoactive intestinal peptide (VIP), which activates CD4+ T cells and ILC2, creating a positive feedback loop that exacerbates asthma. The use of sodium channel inhibitors can alleviate this condition (35). In addition, during IL-25 induced allergic reactions, sensory neurons (Dorsal Root Ganglia, DRG) release Neuromedin U (NMU), which acts on ILC2 via NMUR1, leading to severe allergic responses (36). High expression of CGRP in pulmonary neuroendocrine cells (PNEC) also plays a significant role in asthma. Eosinophil extracellular traps (EETs) released by eosinophils activate PNEC, leading to the release of CGRP and GABA, which further exacerbates the inflammatory response (37). ACh, as the main neurotransmitter of the parasympathetic neurons, acts on pulmonary epithelial cells and immune cells through muscarinic and nicotinic receptors, leading to bronchoconstriction and promoting inflammatory responses. These developments have greatly deepened our insight into the neuroimmune processes involved in the pathogenesis of asthma. As illustrated in **Figure 10**.

This multi-layered knowledge dissemination system effectively promotes the in-depth integration of disciplines such as neuroscience, immunology, and respiratory medicine within this field. As research paradigms continue to innovate and methodologies advance, these core journals will continue to lead the academic development direction in the field of neuroimmunology and pulmonary diseases, driving the ongoing deepening and refinement of this important research area.

Based on literature co−citation analysis (**Figure 6**), This study indicates that the research trajectory in the field of neuroimmunity and pulmonary diseases exhibits a clear phase-dependent evolutionary pattern. In the early phase (2012-2014), core literature focused on the nicotinic cholinergic system and regulatory networks, primarily elucidating the essential role of cholinergic pathways in maintaining pulmonary immune homeostasis. This established a theoretical framework for subsequent investigations into neuro-immune interaction mechanisms, with research largely centered on the pathophysiological processes of classical inflammatory lung diseases. During the middle phase (2016-2019), the field’s focus shifted toward potential analysis and biological abnormalities. Through in-depth analysis of neural signal-mediated regulation of immune cell function, this period propelled the field’s rapid maturation from descriptive phenomenology to mechanistic elucidation. Concurrently, established research directions such as the “sympathetic nerve” remained active, further validating the driving role of neural signals in remodeling the pulmonary inflammatory microenvironment. Following 2020, research centered on COVID-19 infection and air pollution emerged as a new core focus, possibly related to the spike of publications about COVID-19 as well as long COVID during last 6 years, reflecting the field’s deepening exploration of neuro-immune regulatory mechanisms toward the molecular level. Research during this phase both built upon previous theoretical frameworks and advanced the translation of neuroimmune regulatory mechanisms into clinical therapeutic strategies. Notably, the emergence of new keywords such as “sensory neuron” in the timeline suggests ongoing, in-depth exploration of neuro-immune regulatory mechanisms within the field (38). Work by Carly G. K. Ziegler and colleagues has revealed the central role of the sphingosine-1-phosphate (S1P) signaling pathway in lymphocyte trafficking and neuroinflammation, providing a new theoretical basis for targeted therapies in inflammatory diseases (39). This dynamic evolution of research hotspots not only advances our understanding of the interactive network among the nervous system, immunity, and pulmonary diseases but also lays a solid groundwork for the future development of more precisely targeted therapeutic strategies.

From the viewpoint of keyword temporal evolution (**Figure 8C**), Early research concentrated on the interplay between neuroimmune interactions and classic pulmonary diseases, exploring mechanisms such as airway inflammation in asthma, the role of calcitonin gene-related peptide in neural regulation, and the function of the autonomic nervous system in respiratory control, thereby establishing a theoretical foundation for the association between neuroimmunity and pulmonary disorders. As neuroimmunology advanced, research emphasis gradually shifted toward mechanistic depth, with the cholinergic anti-inflammatory pathway, the sympathetic nervous system, and neuropeptides such as substance-P garnering widespread attention for their regulatory roles in inflammatory responses. The outbreak of the COVID-19 pandemic introduced a new research dimension to this field, focusing on neurological complications associated with SARS-CoV-2 infection. This has driven academic exploration across multiple levels—from peripheral neuroimmune mechanisms to central nervous system regulation, and from fundamental science to clinical applications—further refining the theoretical framework linking infection, neuroimmunity, and pulmonary pathology. These advances provide critical theoretical support for the development of novel targeted therapeutic strategies. Overall, the field has evolved from studies of peripheral neuroimmune processes toward central regulation, and from foundational mechanistic work toward clinical application. Future research is expected to further elucidate the neuro–immune–pulmonary interaction network and to provide theoretical underpinnings for the development of novel targeted therapies.

Although this study provides a systematic perspective on understanding the development trends in the neuroimmune-pulmonary disease field through bibliometric analysis, we must cautiously consider the limitations of the research findings. Firstly, the limitations of data sources are noteworthy: this study is based on the Web of Science’s SCI-Expanded database, which primarily includes English-language journal literature. This may exclude significant research published in other languages from the analysis. Secondly, there are inherent constraints in the research methodology: the tools we employed, such as VOSviewer and Citespace, primarily rely on metadata (such as titles, abstracts, and references) for analysis, which may not delve into the deeper semantic content of full texts. This limitation could affect the complete identification of complex interconnections between research themes. Additionally, the inherent latency of bibliometric methods must be considered: on the one hand, emerging high-quality research takes time to accumulate citation frequency; on the other hand, the database update cycle makes it challenging for the latest research findings to be promptly included in the analysis, potentially leading to delays in our judgment of cutting-edge trends. Notwithstanding these limitations, the study’s results still offer useful guidance for scientific planning and future development priorities in this area. Subsequent research might integrate conventional review approaches to examine the principal topics highlighted here more thoroughly.

Our cross-validation with PubMed significantly strengthens the credibility of our findings. Although the two databases differ in absolute publication counts, attributable to Web of Sciences broader interdisciplinary coverage versus PubMed’s emphasis on clinical literature, their core findings are highly consistent. Annual publication trajectories in both datasets follow nearly parallel growth curves and jointly peak in 2021–2022, confirming that the observed expansion of research activity in pulmonary neuroimmunology reflects a genuine field−level trend rather than an artifact of a single database. Keyword comparisons further reveal both the stability of the domain’s focal topics and complementary perspectives: neuroimmunology and pulmonary conditions such as asthma and chronic obstructive pulmonary disease dominate in both sources, while the synchronous attention to SARS−CoV−2–related studies underscores the profound influence of the COVID−19 pandemic on the research agenda. At the same time, the tendency of WoS to emphasize basic mechanisms (e.g., cholinergic anti-inflammatory pathway) and of PubMed to foreground clinical syndromes (e.g., acute respiratory distress syndrome) is congruent with the respective scopes of these databases. This cross−database concordance supports the conclusion that our bibliometric mapping captures the authentic scholarly evolution of the field rather than database−specific bias.

## 5 Conclusion

Bibliometric analysis reveals that the annual number of publications on neuroimmune function in pulmonary diseases has remained relatively stable over the past decade, with a general upward trend indicating promising prospects for neuroimmune research. The United States has emerged as a leading contributor with significant influence in this field. Core research hotspots center on fundamental mechanisms of neuro-immune interaction and lung-brain axis signaling pathways, focusing on neuropeptide and sensory neural pathway regulation in classic pulmonary diseases such as allergic asthma and chronic obstructive pulmonary disease. The COVID-19 pandemic has further propelled comprehensive investigations into the infection-neuroimmunity-pulmonary pathology axis, enriching the field’s research dimensions. Current studies predominantly concentrate on well-characterized neuroimmune mechanisms in inflammatory lung diseases, whereas chronic pulmonary conditions of unknown etiology, such as idiopathic pulmonary fibrosis, have received limited attention regarding their underlying neuroimmune communication. Future research directions are increasingly evident: identifying specific bridges—potentially involving particular immune cell subsets or common inflammatory signals—that mediate bidirectional communication between the lungs and the brain is essential. Furthermore, cross-database validation has corroborated the reliability of these findings and trends. Ultimately, the goal is to identify biomarkers capable of providing early warning for such interdisciplinary diseases, thereby paving the way for precision interventions.

## Data Availability

Publicly available datasets were analyzed in this study. This data can be found here: Web of Science, PubMed.

## References

[1] Megas S Wilbrey-Clark A Maartens A Teichmann SA Meyer KB . Spatial transcriptomics of the respiratory system. Annu Rev Physiol. (2025) 87:447–70. doi: 10.1146/annurev-physiol-022724-105144, PMID: 39353142

[2] Liu R Buttaci DR Sokol CL . Neurogenic inflammation and itch in barrier tissues. Semin Immunol. (2025) 77:101928. doi: 10.1016/j.smim.2024.101928, PMID: 39798211 PMC11893243

[3] Godinho-Silva C Cardoso F Veiga-Fernandes H . Neuro-immune cell units: A new paradigm in physiology. Annu Rev Immunol. (2019) 37:19–46. doi: 10.1146/annurev-immunol-042718-041812, PMID: 30379595

[4] Blake KJ Jiang XR Chiu IM . Neuronal regulation of immunity in the skin and lungs. Trends Neurosci. (2019) 42:537–51. doi: 10.1016/j.tins.2019.05.005, PMID: 31213389 PMC6661013

[5] Azzoni R Perdijk O Harris NL Marsland BJ . Neuroimmunology of the lung. Annu Rev Immunol. (2024) 42:57–81. doi: 10.1146/annurev-immunol-083122-042512, PMID: 37989144

[6] Brabenec L Gupta S Eichwald T Rafei M Talbot S . Decoding the neuroimmune axis in the atopic march: mechanisms and implications. Curr Opin Immunol. (2024) 91:102507. doi: 10.1016/j.coi.2024.102507, PMID: 39579588

[7] Xu A Liu Y Li S Zhan C Cheng Y Zhang C . Global burden of major chronic respiratory diseases among older adults aged 55 and above from 1990 to 2021: Changes, challenges, and predictions amid the pandemic. PloS One. (2025) 20:e0329283. doi: 10.1371/journal.pone.0329283, PMID: 40749041 PMC12316243

[8] Global age-sex-specific all-cause mortality and life expectancy estimates for 204 countries and territories and 660 subnational locations, 1950-2023: a demographic analysis for the Global Burden of Disease Study 2023. Lancet. (2025) 406:1731–810. doi: 10.1016/S0140-6736(25)01330-3, PMID: 41092927 PMC12535839

[9] Wang X Li X Dong T Yu W Jia Z Hou Y . Global biomarker trends in triple-negative breast cancer research: a bibliometric analysis. Int J Surg. (2024) 110:7962–83. doi: 10.1097/JS9.0000000000001799, PMID: 38857504 PMC11634138

[10] Qu X Wang Q Zhu F Liang H Long Z Wu Y . Research hotspots and trends in immunotherapy for cholangiocarcinoma: a bibliometric analysis (2014-2023). Front Immunol. (2024) 15:1436315. doi: 10.3389/fimmu.2024.1436315, PMID: 39660136 PMC11628549

[11] Lv M Feng Y Zeng S Zhang Y Shen W Guan W . A bibliometrics analysis based on the application of artificial intelligence in the field of radiotherapy from 2003 to 2023. Radiat Oncol. (2024) 19:157. doi: 10.1186/s13014-024-02551-1, PMID: 39529129 PMC11552138

[12] Arruda H Silva ER Lessa M Proença DJ Bartholo R . VOSviewer and bibliometrix. J Med Libr Assoc. (2022) 110:392–5. doi: 10.5195/jmla.2022.1434, PMID: 36589296 PMC9782747

[13] van Eck NJ Waltman L . Software survey: VOSviewer, a computer program for bibliometric mapping. Scientometrics. (2010) 84:523–38. doi: 10.1007/s11192-009-0146-3, PMID: 20585380 PMC2883932

[14] Liu S Sun Y-P Gao X-L Sui Y . Knowledge domain and emerging trends in Alzheimer’s disease: a scientometric review based on CiteSpace analysis. Neural Regener Res. (2019) 14:1643–50. doi: 10.4103/1673-5374.255995, PMID: 31089065 PMC6557102

[15] Deng P Wang S Sun X Qi Y Ma Z Pan X . Global trends in research of gouty arthritis over past decade: A bibliometric analysis. Front Immunol. (2022) 13:910400. doi: 10.3389/fimmu.2022.910400, PMID: 35757713 PMC9229989

[16] Hirsch JE . An index to quantify an individual’s scientific research output. Proc Natl Acad Sci U.S.A. (2005) 102:16569–72. doi: 10.1073/pnas.0507655102, PMID: 16275915 PMC1283832

[17] Roldan-Valadez E Salazar-Ruiz SY Ibarra-Contreras R Rios C . Current concepts on bibliometrics: a brief review about impact factor, Eigenfactor score, CiteScore, SCImago Journal Rank, Source-Normalised Impact per Paper, H-index, and alternative metrics. Ir J Med Sci. (2019) 188:939–51. doi: 10.1007/s11845-018-1936-5, PMID: 30511320

[18] Khazaneha M Osareh F Shafiee K . Trend linking of multiple system atrophy: A scientometric study. Endocr Metab Immune Disord Drug Targets. (2021) 21:700–10. doi: 10.2174/1871530320666200607194810, PMID: 32515315

[19] Chen C Ibekwe-SanJuan F Hou J . The structure and dynamics of cocitation clusters: A multiple-perspective cocitation analysis. J Am Soc Inf Sci Technol. (2010) 61:1386–409. doi: 10.1002/asi.21309, PMID: 41767589

[20] Yuan R Tan Y Sun P-H Qin B Liang Z . Emerging trends and research foci of berberine on tumor from 2002 to 2021: A bibliometric article of the literature from WoSCC. Front Pharmacol. (2023) 14:1122890. doi: 10.3389/fphar.2023.1122890, PMID: 36937842 PMC10021304

[21] Chen Y Chen Y Tan S Zheng Y Liu S Zheng T . Visual analysis of global research on immunotherapy for gastric cancer: A literature mining from 2012 to 2022. Hum Vaccin Immunother. (2023) 19:2186684. doi: 10.1080/21645515.2023.2186684, PMID: 37017186 PMC10088978

[22] D T NH K S Ma H C Z . Population of sensory neurons essential for asthmatic hyperreactivity of inflamed airways. Proc Natl Acad Sci United States America. (2014) 111:9. doi: 10.1073/pnas.1411032111, PMID: 25049382 PMC4128113

[23] Desforges M Le Coupanec A Dubeau P Bourgouin A Lajoie L Dubé M . Human coronaviruses and other respiratory viruses: underestimated opportunistic pathogens of the central nervous system? Viruses. (2019) 12:14. doi: 10.3390/v12010014, PMID: 31861926 PMC7020001

[24] Ea T Jn K SH . Are we facing a crashing wave of neuropsychiatric sequelae of COVID-19? Neuropsychiatric symptoms and potential immunologic mechanisms. Brain behavior Immun. (2020) 87:10. doi: 10.1016/j.bbi.2020.04.027, PMID: 32298803 PMC7152874

[25] Wallrapp A Riesenfeld SJ Burkett PR Abdulnour R-EE Nyman J Dionne D . The neuropeptide NMU amplifies ILC2-driven allergic lung inflammation. Nature. (2017) 549:351–6. doi: 10.1038/nature24029, PMID: 28902842 PMC5746044

[26] Klose CSN Mahlakõiv T Moeller JB Rankin LC Flamar A-L Kabata H . The neuropeptide Neuromedin U stimulates innate lymphoid cells and type 2 inflammation. Nature. (2017) 549:282–6. doi: 10.1038/nature23676, PMID: 28869965 PMC6066372

[27] Borovikova LV Ivanova S Zhang M Yang H Botchkina GI Watkins LR . Vagus nerve stimulation attenuates the systemic inflammatory response to endotoxin. Nature. (2000) 405:458–62. doi: 10.1038/35013070, PMID: 10839541

[28] Wang H Yu M Ochani M Amella CA Tanovic M Susarla S . Nicotinic acetylcholine receptor alpha7 subunit is an essential regulator of inflammation. Nature. (2003) 421:384–8. doi: 10.1038/nature01339, PMID: 12508119

[29] Bouffette S Botez I De Ceuninck F . Targeting galectin-3 in inflammatory and fibrotic diseases. Trends Pharmacol Sci. (2023) 44:519–31. doi: 10.1016/j.tips.2023.06.001, PMID: 37391294

[30] B G Dd G K M P A Ea M H D . Asthma innovations from the first International Collaborative Asthma Network forum. ERJ Open Res. (2023) 9:13. doi: 10.1183/23120541.00090-2023, PMID: 37260461 PMC10227632

[31] Khoury M Faiz SA Sheshadri A . Immune checkpoint inhibitor-associated pneumonitis: focus on diagnosis and underlying mechanisms. Curr Opin Pulm Med. (2025) 31:335–43. doi: 10.1097/MCP.0000000000001175, PMID: 40265506

[32] Veiga-Fernandes H Mucida D . Neuro-immune interactions at barrier surfaces. Cell. (2016) 165:801–11. doi: 10.1016/j.cell.2016.04.041, PMID: 27153494 PMC4871617

[33] Jayasooriya SM Devereux G Soriano JB Singh N Masekela R Mortimer K . Asthma: epidemiology, risk factors, and opportunities for prevention and treatment. Lancet Respir Med. (2025) 13:725–38. doi: 10.1016/S2213-2600(24)00383-7, PMID: 40684789

[34] Ai C M B Md E Bf B D del C M D . A sensory neuronal ion channel essential for airway inflammation and hyperreactivity in asthma. Proc Natl Acad Sci United States America. (2009) 106:13. doi: 10.1073/pnas.0900591106, PMID: 19458046 PMC2684498

[35] S T Re A Pr B S L Sj C Ma P . Silencing nociceptor neurons reduces allergic airway inflammation. Neuron. (2015) 87. doi: 10.1016/j.neuron.2015.06.007, PMID: 26119026 PMC4506220

[36] A W Sj R Pr B Re A J N D D . Erratum: The neuropeptide NMU amplifies ILC2-driven allergic lung inflammation. Nature. (2017) 551. doi: 10.1038/nature24480, PMID: 29143821

[37] Eosinophil extracellular traps drive asthma progression through neuro-immune signals - PubMed. Available online at: https://vpnlib.cmc.edu.cn/https/77726476706e69737468656265737421e0e243912234265e7d0a80e296592e7bb7d62ae2c192eb/34616019/ (Accessed November 13, 2025). 10.1038/s41556-021-00762-2 34616019

[38] B B Ya A A S A L-R T V K T . Differential effects of lipopolysaccharide on mouse sensory TRP channels. . Cell calcium. (2018) 73:15. doi: 10.1016/j.ceca.2018.04.004, PMID: 29689522

[39] Huang S Ziegler CGK Austin J Mannoun N Vukovic M Ordovas-Montanes J . Lymph nodes are innervated by a unique population of sensory neurons with immunomodulatory potential. Cell. (2021) 184:441–459.e25. doi: 10.1016/j.cell.2020.11.028, PMID: 33333021 PMC9612289

